# Assessment of Intratumor Heterogeneity in Parametric Dynamic Contrast-Enhanced MR Images: A Comparative Study of Novel and Established Methods

**DOI:** 10.3389/fonc.2021.722773

**Published:** 2021-09-21

**Authors:** Jon-Vidar Gaustad, Einar K. Rofstad

**Affiliations:** Group of Radiation Biology and Tumor Physiology, Department of Radiation Biology, Institute for Cancer Research, Oslo University Hospital, Oslo, Norway

**Keywords:** intratumor heterogeneity, DCE-MRI, spatial gradient method, histogram analysis, Haralick features, sunitinib, pancreatic ductal adenocarcinoma

## Abstract

Intratumor heterogeneity is associated with aggressive disease and poor survival rates in several types of cancer. A novel method for assessing intratumor heterogeneity in medical images, named the spatial gradient method, has been developed in our laboratory. In this study, we measure intratumor heterogeneity in *K* ^trans^ maps derived by dynamic contrast-enhanced magnetic resonance imaging using the spatial gradient method, and we compare the performance of the novel method with that of histogram analyses and texture analyses using the Haralick method. *K* ^trans^ maps of 58 untreated and sunitinib-treated pancreatic ductal adenocaricoma (PDAC) xenografts from two PDAC models were investigated. Intratumor heterogeneity parameters derived by the spatial gradient method were sensitive to tumor line differences as well as sunitinib-induced changes in intratumor heterogeneity. Furthermore, the parameters provided additional information to the median value and were not severely affected by imaging noise. The parameters derived by histogram analyses were insensitive to spatial heterogeneity and were strongly correlated to the median value, and the Haralick features were severely influenced by imaging noise and did not differentiate between untreated and sunitinib-treated tumors. The spatial gradient method was superior to histogram analyses and Haralick features for assessing intratumor heterogeneity in *K* ^trans^ maps of untreated and sunitinib-treated PDAC xenografts, and can possibly be used to assess intratumor heterogeneity in other medical images and to evaluate effects of other treatments as well.

## Introduction

Advanced tumors can show substantial variation in structure and function within individual lesions (1, 2). This intratumor heterogeneity arises through complex genetic, epigenetic, and protein modifications in response to a heterogeneous environmental pressure, and it allows tumors to develop significant adaptive capability (3). Consequently, intratumor heterogeneity is believed to be an important cause of treatment resistance, and has been shown to be associated with aggressive disease and poor prognosis in several types of cancer (1–3).

The abnormal tumor vasculature plays a key role in establishing the heterogeneous environmental pressure (4). The vascular abnormalities include heterogeneous vessel density, aberrant vessel diameters, and tortuous and elongated vessels, and these collectively impair blood flow and oxygen supply (4–6). Most tumors thus develop regions with hypoxic tissue, and the same tumors can also show regions with highly elevated vessel density (vascular hot spots). Interestingly, both tumor hypoxia and high vessel density in vascular hot spots has been associated with increased incidence of metastases and poor prognosis (7, 8).

Dynamic contrast-enhanced magnetic resonance imaging (DCE-MRI) has been used to determine the location and size of tumor lesions, and have also been used to characterize the tumor microenvironment by providing functional information such as tumor blood flow, vessel permeability, and tumor hypoxia (9, 10). This information has been used for diagnosis and prognosis, and by repeating imaging sessions, longitudinal information on disease development as well as effects of treatments have been obtained (10, 11). DCE-MRI-derived parametric images contain spatial information, but most of the studies have only reported average or median values (1, 12). In doing so, information about the intratumor heterogeneity and the spatial complexity of the tumors has been discarded.

However, some studies have quantified intratumor heterogeneity in parametric DCE-MR images, and the most commonly used methods are based on histogram analyses or texture analyses using the Haralick method (13). The histogram analyses are relatively straight forward and can provide important information on the range of values (13–16), but do not consider the spatial location of the voxels (1). The Haralick method is based on calculating a co-occurrence matrix from which up to 13 Haralick features can be extracted (17, 18). The Haralick features consider the spatial location of voxels, but the biological interpretation of the features is unclear and it is not obvious how the features are influenced by imaging noise.

An ideal method for quantifying intratumor heterogeneity should be objective and calculate parameters from individual voxel values. The method should provide additional information to the median value, and should not be confounded by imaging noise. Furthermore, the method should be able to quantify intratumor heterogeneity parameters in both untreated and treated tumors, and should be sensitive to treatment-induced changes.

In our laboratory, we have developed a new method for quantifying intratumor heterogeneity in medical images of tumor tissue independent of cancer type. The method measures spatial gradients in parametric images, and has been named the spatial gradient method. The aim of this study was to evaluate the performance of the spatial gradient method in DCE-MRI-derived images of *K* ^trans^ [the volume transfer constant of the contrast agent (19)], and to compare the method with histogram analyses and the Haralick method. Furthermore, we chose to explore the method in pancreatic ductal adenocarcinoma (PDAC), because patients with PDAC have particularly poor prognosis (20–22). We used *K* ^trans^ maps of untreated and sunitinib-treated tumors of two pancreatic PDAC xenograft models obtained in a previous study (23), and we quantified heterogeneity in the *K* ^trans^ maps by using the spatial gradient method, histogram analyses, and the Haralick method.

## Materials and Methods

### Data Sets

*K*^trans^ maps of untreated and sunitinib-treated BxPC-3 and Panc-1 PDAC xenografts from a previous study by Wegner et al. (23) were used in the current study. Twenty-eight data sets of BxPC-3 tumors (15 untreated and 13 sunitinib-treated tumors) and thirty data sets of Panc-1 tumors (20 untreated and 10 sunitinib-treated tumors) were included. The PDAC models, the sunitinib treatment, and the DCE-MRI protocol have been described in detail previously (23). Briefly, BxPC-3 and Panc-1 tumors (American Type Culture Collection, VA, USA) were initiated in the left *quadriceps femoris* of BALB/c *nu*/*nu* mice, and were included in the experiments when the tumors were vascularized and had grown to a size of 200-1200 mm^3^. Tumor-bearing mice were treated with 40 mg/kg/day sunitinib (LC Laboratories, Woburn, MA, USA) or vehicle for 4 days by oral administration using a gavage, and were subjected to DCE-MRI one day after the last sunitinib dose.

DCE-MRI was performed on a preclinical 7-T scanner (Bruker Biospin, Ettlingen, Germany) by using Gd-DOTA (Dotarem, Guerbet, Paris, France) as contrast agent. The tumors were positioned in the isocenter of the magnet and were imaged with axial slices covering the entire volume. Dynamic T_1_-weighted images were recorded at a temporal resolution of 14.8 s and a voxel size of 0.23 × 0.23 × 1.0 mm^3^. Gd-DOTA concentrations were calculated from the T_1_-weighted images by using T_1_ maps recorded before Gd-DOTA injection as detailed elsewhere (24). For each voxel, numerical values of *K* ^trans^ (the volume transfer constant of Gd-DOTA) were determined by using Tofts generalized pharmacokinetic model (19) and the arterial input function reported by Benjaminsen et al. (25). The mice were given gas anesthesia (∼4.0% Sevofluran in O2; Baxter, IL, USA) at a flow rate of 0.5 l/min and were fixed to the bore during imaging. The body core temperature was kept at 37°C by automated hot air flow regulation, and the gas anesthesia was adjusted manually to maintain a stable respiration rate.

### Histogram Analyses

*K*^trans^ histograms were produced by including individual voxel values of the entire tumor volume. The median, the variance, the histogram width, and the histogram skewness were calculated from *K* ^trans^ histograms by using in-house-made software developed in Matlab (MathWorks, Natick, MA, USA). The histogram width was defined as the range of values in the histogram with a frequency > 10% of the maximum frequency. The parameters variance and histogram skewness were calculated from the values within the same range.

### Haralick Features

Gray level co-occurrence matrices (GLCM) were calculated from *K* ^trans^ maps of the central axial section of each tumor using 8 gray levels, a distance of 1 mm, and the directions 0° and 90°. The Haralick features Contrast, Energy, Homogeneity, and Correlation were extracted from the GLCM as described in detail by Haralick et al. (17). A description of how the Haralick features should be interpreted has been provided by Vribik et al. (18). Briefly, Contrast is a measure of the variation in intensity between a voxel and its neighbor voxels and is 0 for a perfectly homogeneous image. Energy (also known as uniformity or the angular second moment) measures the sum of the squared elements in the GLCM and ranges from 0 to 1 (1 for a perfectly homogeneous image). Homogeneity measures how close the distribution of the GLCM elements is to the GLCM diagonal and is close to 1 when only a few gray levels are present. Correlation measures how correlated a voxel is to its neighbor and is 1 or -1 for a perfect positively or negatively correlated image and 0 for a perfectly homogeneous image. The GLCM and the Haralick features were computed by using the functions *graycomatrix* and *graycoprops* in the Matlab Image Processing Toolbox.

### The Spatial Gradient Method

Gradients in *K* ^trans^ maps (Δ*K* ^trans^ *versus* distance) were calculated from peripheral ROIs and from high value ROIs in the central axial section of each tumor. The peripheral ROIs consisted of a 3-voxel thick doughnut in the tumor periphery, whereas the high value ROIs consisted of the voxels with *K* ^trans^ values greater than the 75^th^ percentile. For each voxel in the peripheral or the high value ROI, the difference in *K* ^trans^ (Δ*K* ^trans^) between the voxel and a neighbor tumor voxel at a certain distance was measured. Δ*K* ^trans^ *versus* distance was measured in two directions (0° and 90°) and averaged for the two directions. Curves showing PRΔ*K* ^trans^ *versus* distance were produced by averaging Δ*K* ^trans^ *versus* distance of all individual voxels in the peripheral ROIs (PR denotes peripheral ROI), and curves showing HVRΔ*K* ^trans^ *versus* distance were produced by averaging Δ*K* ^trans^ *versus* distance of all individual voxels in the high value ROIs (HVR denotes high value ROI). PRΔ*K* ^trans^ at a distance of 5 mm $\left(\text{PR}\Delta K_{5\text{ mm}}^{\text{trans}}\right)$and HVRΔ*K* ^trans^ at a distance of 2 mm $\left(\text{HVR}\Delta K_{2\text{ mm}}^{\text{trans}}\right)$ were extracted from the curves and used as parameters for intratumor heterogeneity.

### Statistical Analysis

Statistical comparisons of data were carried out by the Student’s t test when the data complied with the conditions of normality and equal variance. Under other conditions, comparisons were done by nonparametric analysis using the Mann-Whitney rank sum test. The Kolmogorov-Smirnov method was used to test for normality, and the Levene’s test was used to test for equal variance. Probability values of *P <* 0.05, determined from two-sided tests, were considered significant. The Pearson product moment correlation test was used to search for correlation between parameters. Curves were fitted to the data by regression analysis. The statistical analysis was performed by using the SigmaStat statistical software (SPSS Science, Chicago, IL, USA).

## Results

**Figure 1:**
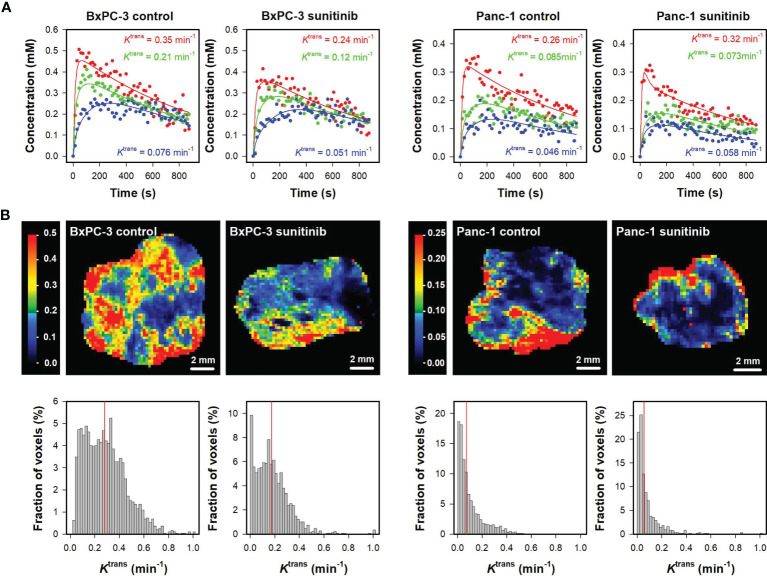
**(A)** Plots of Gd-DOTA concentration *versus* time and the corresponding pharmacokinetic model fits for individual voxels in representative untreated and sunitinib-treated BxPC-3 tumors and representative untreated and sunitinib-treated Panc-1 tumors. The *K* ^trans^ values of the voxels are labeled in the panels. **(B)** *K* ^trans^ maps and histograms of the representative untreated and sunitinib-treated BxPC-3 tumors and the representative untreated and sunitinib-treated Panc-1 tumors. *K* ^trans^ scales are given by the color bars, and median *K* ^trans^ values are indicated by red vertical lines in the *K* ^trans^ histograms.

**Figure 1A** shows plots of Gd-DOTA concentration *versus* time and the corresponding pharmacokinetic model fits for individual voxels in representative untreated and sunitinib-treated BxPC-3 tumors and in representative untreated and sunitinib-treated Panc-1 tumors. The figure illustrates that individual voxels differed substantially in the uptake of Gd-DOTA, and good model fits were obtained in voxels with both high and low uptake. *K* ^trans^ maps and histograms of the representative tumors are shown in **Figure 1B**. The intratumor heterogeneity in *K* ^trans^ was substantial in both models, and the range of *K* ^trans^ values appeared to be higher in BxPC-3 tumors than in Panc-1 tumors. Furthermore the highest *K* ^trans^ values were generally found in peripheral regions in both models.

**Figure 2:**
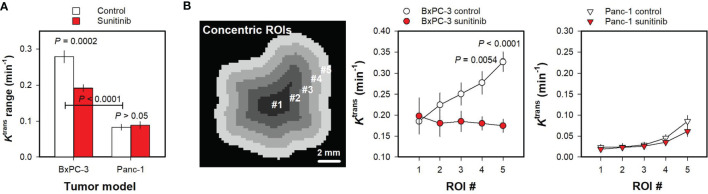
**(A)***K*^trans^ range in untreated and sunitinib-treated BxPC-3 tumors and in untreated and sunitinib-treated Panc-1 tumors. The *K* ^trans^ range represents the difference between the 25^th^ and the 75^th^ percentile and was calculated for each tumor. Columns and bars, mean ± SEM of 10-20 tumors. **(B)** Illustration of five concentric regions of interest (ROI #1-5) in the representative untreated BxPC-3 tumor shown in **Figure 1**, and median *K* ^trans^ in concentric ROIs in untreated and sunitinib-treated BxPC-3 tumors and in untreated and sunitinib-treated Panc-1 tumors. The concentric ROIs are bounded by lines drawn at distances of nR/5 from the tumor center, where R is tumor radius and n is ROI number (#1-5). Sunitinib-treated BxPC-3 tumors showed lower *K* ^trans^ values than untreated BxPC-3 tumors in ROI #4 (*P* =0.0054) and ROI #5 (*P* < 0.0001), whereas the *K* ^trans^ values in the ROIs #1-3 did not differ between the treatment groups (*P* > 0.05). Untreated and sunitinib-treated Panc-1 tumors did not differ in *K* ^trans^ in any of the concentric ROIs (ROIs #1-5: *P* > 0.05). Points and bars, mean ± SEM of 10-20 tumors.

To quantify these qualitative observations and determine ground truth, the difference between the 25^th^ and the 75^th^ *K* ^trans^ percentile was measured and used as parameter for *K* ^trans^ range, and *K* ^trans^ maps were divided in concentric ROIs to search for radial heterogeneity (**Figure 2**). Untreated BxPC-3 tumors showed a higher *K* ^trans^ range than untreated Panc-1 tumors (**Figure 2A**; *P* < 0.0001). Furthermore, sunitinib-treated BxPC-3 tumors showed a lower *K* ^trans^ range than untreated BxPC-3 tumors (**Figure 2A**; *P* = 0.0002), whereas untreated and sunitinib-treated Panc-1 tumors did not differ in *K* ^trans^ range (**Figure 2A**; *P* > 0.05). Untreated tumors of both models showed radial heterogeneity in *K* ^trans^, i.e. *K* ^trans^ was low in central tumor regions and increased gradually towards the periphery (**Figure 2B**). The radial heterogeneity was more pronounced in untreated BxPC-3 tumors than in untreated Panc-1 tumors. Sunitinib-treated BxPC-3 tumors showed lower *K* ^trans^ values than untreated BxPC-3 tumors in the peripheral ROIs (**Figure 2B**; ROI #4: *P* =0.0054; ROI#5: *P* < 0.0001), whereas the *K* ^trans^ values in the central ROIs did not differ between the treatment groups (**Figure 2B**; ROIs #1-3: *P* > 0.05). This finding implied that the sunitinib treatment affected the peripheral regions and was ineffective in the central regions, and consequently radial heterogeneity in *K* ^trans^ was not observed in sunitinib-treated BxPC-3 tumors. Untreated and sunitinib-treated Panc-1 tumors did not differ in *K* ^trans^ in any of the concentric ROIs (**Figure 2B**; ROIs #1-5: *P* > 0.05), and thus showed similar radial heterogeneity.

**Figure 3:**
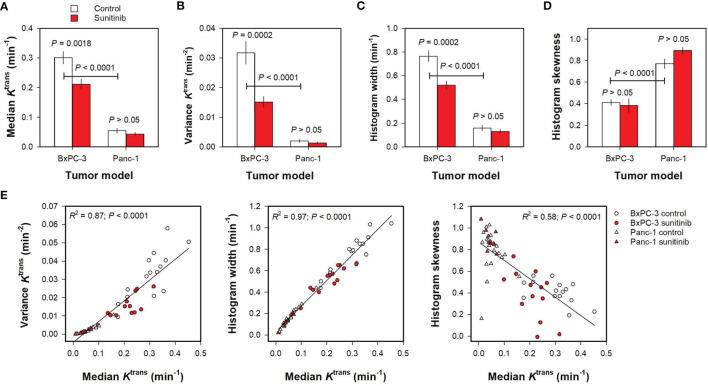
**(A–D)** Median *K* ^trans^, variance *K* ^trans^, *K* ^trans^ histogram width, and *K* ^trans^ histogram skewness in untreated and sunitinib-treated BxPC-3 tumors and in untreated and sunitinib-treated Panc-1 tumors. Columns and bars, mean ± SEM of 10-20 tumors. **(E)** Variance *K* ^trans^, *K* ^trans^ histogram width, and *K* ^trans^ histogram skewness *versus* median *K* ^trans^ in untreated and sunitinib BxPC-3 and in untreated and sunitinib-treated Panc-1 tumors. Points, individual tumors; Curves, linear regression lines.

Parameters derived by histogram analyses are shown in **Figure 3**. Untreated BxPC-3 tumors showed higher median *K* ^trans^, variance *K* ^trans^, and *K* ^trans^ histogram width than untreated Panc-1 tumors (**Figures 3A–C**; *P <*0.0001), and untreated Panc-1 tumors showed higher histogram skewness and thus more asymmetric *K* ^trans^ histograms than untreated BxPC-3 tumors (**Figure 3D**; *P* < 0.0001). Furthermore, sunitinib-treated BxPC-3 tumors showed lower median *K* ^trans^, variance *K* ^trans^, and *K* ^trans^ histogram width than untreated BxPC-3 tumors (**Figures 3A–C**; *P* < 0.002), whereas untreated and sunitinib-treated Panc-1 tumors did not differ in median *K* ^trans^, variance *K* ^trans^, or *K* ^trans^ histogram width (**Figures 3A–C**; *P* > 0.05). Untreated and sunitinib-treated tumors did not differ in *K* ^trans^ histogram skewness in any of the models implying that the treatment did not alter the shape of the *K* ^trans^ histograms (**Figure 3D**; *P* > 0.05). Strong correlations were found between variance *K* ^trans^ and median *K* ^trans^, between *K* ^trans^ histogram width and median *K* ^trans^, and between *K* ^trans^ histogram skewness and median *K* ^trans^ for individual tumors (**Figure 3E**; *R* ^2^ = 0.87, 0.97 and 0.58; *P* < 0.0001), questioning whether the variance, the histogram width, and the histogram skewness provided additional information to the median value.

**Figure 4:**
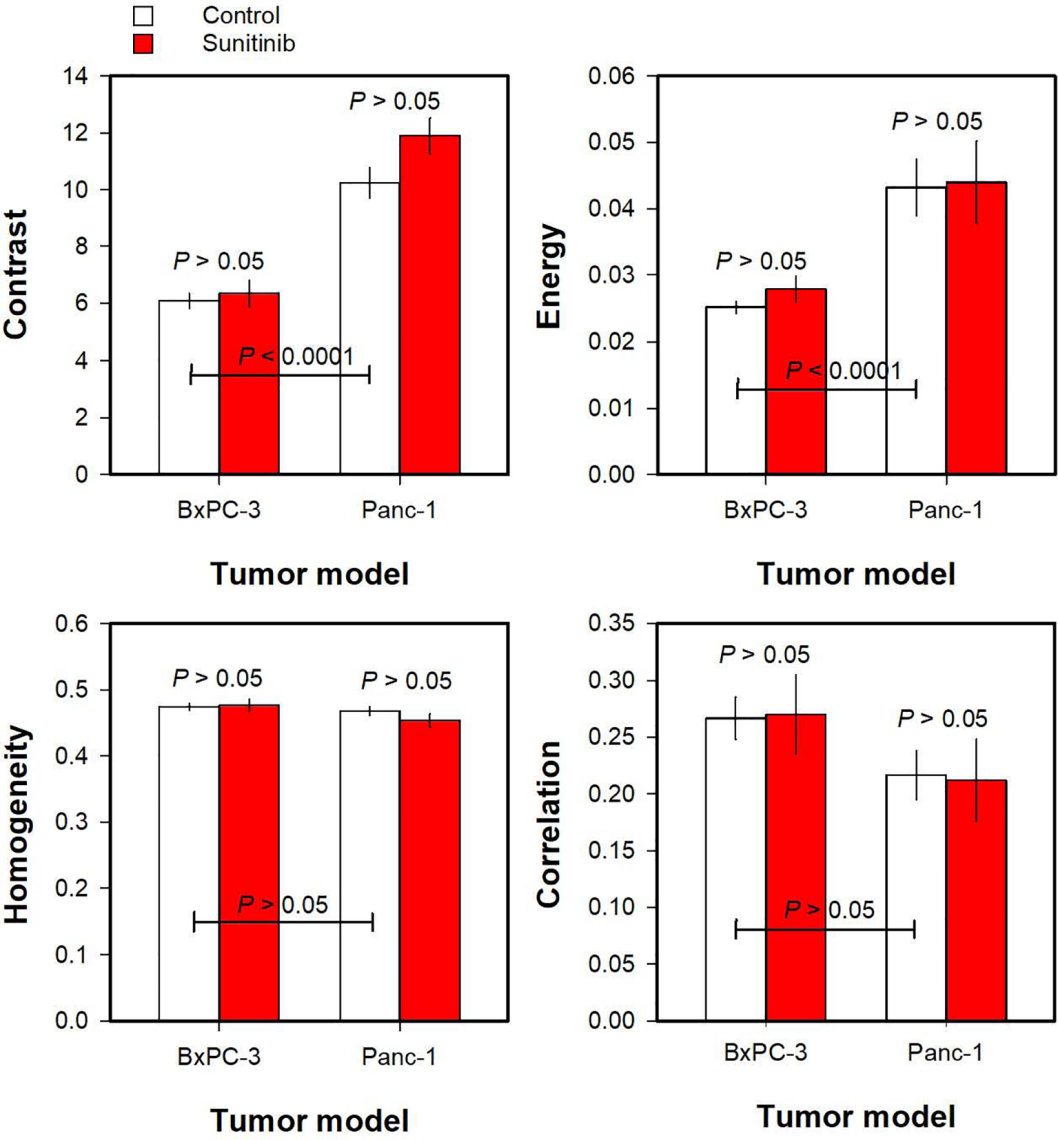
The Haralick features Contrast, Energy, Homogeneity, and Correlation derived from *K* ^trans^ maps of untreated and sunitinib-treated BxPC-3 tumors and untreated and sunitinib-treated Panc-1 tumors. Columns and bars, mean ± SEM of 10-20 tumors.

Haralick features derived from *K* ^trans^ maps are shown in **Figure 4**. Untreated Panc-1 tumors showed higher Contrast and Energy than untreated BxPC-3 tumors (**Figure 4**; *P* < 0.0001), whereas the Haralick features Homogeneity and Correlation did not differ between the PDAC models (**Figure 4**; *P* > 0.05). Furthermore, none of the Haralick features differed between untreated and sunitinib-treated tumors (**Figure 4**; *P* > 0.05).

**Figure 5:**
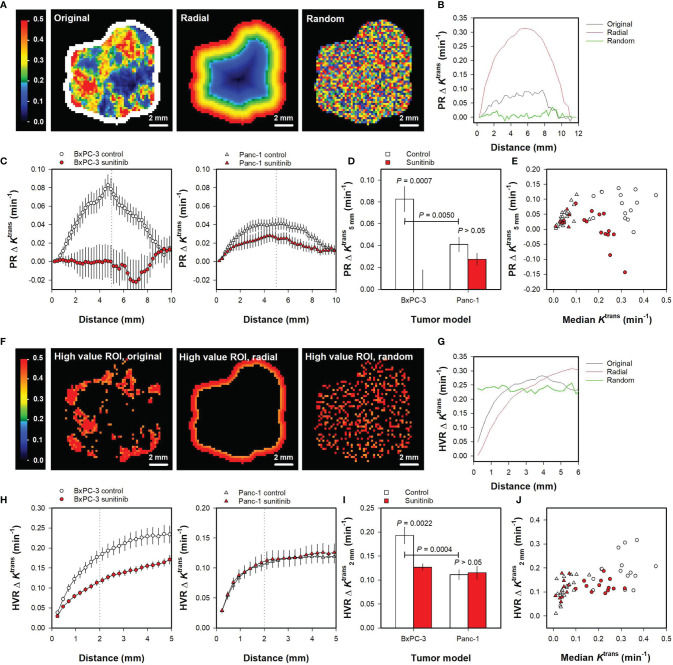
**(A)***K*^trans^ maps of the representative untreated BxPC-3 tumor shown in **Figure 1**. The *K* ^trans^ voxel values are shown in the original geometry and rearranged in a perfectly radial geometry and a random geometry. A peripheral region of interest (ROI) is shown in white color, and the *K* ^trans^ scale is given by the color bar. **(B)** The *K* ^trans^ gradient measured from the peripheral ROI (PRΔ*K* ^trans^ *versus* distance) for the untreated BxPC-3 tumor in the original, the perfectly radial, and the random geometry. **(C)** PRΔ*K* ^trans^ *versus* distance for untreated and sunitinib-treated BxPC-3 tumors and for untreated and sunitinib-treated Panc-1 tumors. Points and bars, mean ± SEM of 10-20 tumors. Dotted line indicate a distance of 5 mm. **(D)** PRΔ*K* ^trans^ measured at a distance of 5 mm ($\text{PR}\Delta K_{5\text{ mm}}^{\text{trans}}$) in untreated and sunitinib-treated BxPC-3 tumors and in untreated and sunitinib-treated Panc-1 tumors. Columns and bars, mean ± SEM of 10-20 tumors. **(E)** $\text{PR}\Delta K_{5\text{ mm}}^{\text{trans}}$ *versus* median *K* ^trans^ in untreated and sunitinib-treated BxPC-3 and in untreated and sunitinib-treated Panc-1 tumors. Points, individual tumors. **(F)** High value ROIs consisting of voxels with *K* ^trans^ values greater that the 75^th^ percentile for the representative untreated BxPC-3 tumor where the tumor voxels are arranged in the original geometry and rearranged in a perfectly radial and random geometry. The *K* ^trans^ scale is given by the color bar. **(G)** The *K* ^trans^ gradient measured from the high value ROIs (HVRΔ*K* ^trans^ *versus* distance) for the untreated BxPC-3 tumor in the original, the perfectly radial, and the random geometry. **(H)** HVRΔ*K* ^trans^ *versus* distance for untreated and sunitinib-treated BxPC-3 tumors and for untreated and sunitinib-treated Panc-1 tumors. Points and bars, mean ± SEM of 10-20 tumors. Dotted lines indicate a distance of 2 mm. **(I)** HVRΔ*K* ^trans^ measured at a distance of 2 mm ($\text{HVR}\Delta K_{2\text{ mm}}^{\text{trans}}$) in untreated and sunitinib-treated BxPC-3 tumors and in untreated and sunitinib-treated Panc-1 tumors. Columns and bars, mean ± SEM of 10-20 tumors. **(J)** $\text{HVR}\Delta K_{2\text{ mm}}^{\text{trans}}$ *versus* median *K* ^trans^ in untreated and sunitinib-treated BxPC-3 and in untreated and sunitinib-treated Panc-1 tumors. Points, individual tumors.

Finally, the spatial gradient method was used to quantify intratumor heterogeneity in the PDAC xenografts. **Figures 5A, B** illustrate the spatial gradient method when using a peripheral ROI. **Figure 5A** shows *K* ^trans^ maps of the representative untreated BxPC-3 tumor where the tumor voxels are arranged in the original geometry and rearranged in a perfectly radial geometry and a random geometry. *K* ^trans^ gradients were measured from voxels in the peripheral ROI (shown in white color in the original *K* ^trans^ map), and curves showing PRΔ*K* ^trans^ *versus* distance were produced by averaging the *K* ^trans^ gradients of all voxels in the peripheral ROI. **Figure 5B** illustrates that the three geometries showed different PRΔ*K* ^trans^ *versus* distance curves. For the perfectly radial geometry, voxels close to the peripheral ROI were similar to the voxels in the peripheral ROI (Δ*K* ^trans^ ≈ 0), and voxels in the central regions had lower *K* ^trans^ values than the voxels in the peripheral ROI (high Δ*K* ^trans^). Thus PRΔ*K* ^trans^ started at 0 (distance = 0 mm), increased towards a maximum in the tumor center (distance = 5.5 mm), and decreased towards the periphery on the opposite side (distance = 11 mm). For the random geometry, some voxels had higher values and others had lower values than the voxels in the peripheral ROI, and, consequently, PRΔ*K* ^trans^ was close to 0 for all distances. The PRΔ*K* ^trans^ *versus* distance curve for the original geometry was similar to the PRΔ*K* ^trans^ *versus* distance curve for the perfectly radial geometry, but the radial trend was less pronounced and superimposed by some variation.

**Figure 5C** shows PRΔ*K* ^trans^ *versus* distance curves for all untreated and sunitinib-treated BxPC-3 tumors and for all untreated and sunitinib-treated Panc-1 tumors included in the study. The figure illustrates that untreated BxPC-3 tumors showed a radial heterogeneity whereas sunitinib-treated BxPC-3 tumors did not. Also Panc-1 tumors showed a radial heterogeneity, and this trend was more pronounced for untreated than for sunitinib-treated tumors. To quantify these observations, the parameter $\text{PR}\Delta K_{5\text{ mm}}^{\text{trans}}$ was extracted from the curves. $\text{PR}\Delta K_{5\text{ mm}}^{\text{trans}}$ was higher for untreated BxPC-3 tumors than for untreated Panc-1 tumors (**Figure 5D**; *P* = 0.0050), and higher for untreated BxPC-3 tumors than for sunitinib-treated BxPC-3 tumors (**Figure 5D**; *P* = 0.0007). Sunitinib-treated Panc-1 tumors tended to have a lower $\text{PR}\Delta K_{5\text{ mm}}^{\text{trans}}$ than untreated Panc-1 tumors but this difference did not reach statistical significance (**Figure 5D**; *P* > 0.05). No correlations were found between $\text{PR}\Delta K_{5\text{ mm}}^{\text{trans}}$ and median *K* ^trans^ for individual tumors (**Figure 5E**), implying that this intratumor heterogeneity parameter provided additional information to the median value.

*K*^trans^ gradients were also measured from voxels in high value ROIs (i.e. voxels with *K* ^trans^ > 75^th^ percentile). **Figures 5F, G** illustrate the spatial gradient method when using high value ROIs. **Figure 5F** shows the high value ROIs of the representative BxPC-3 tumor when the tumor voxels are arranged in the original geometry, and when the tumor voxels are rearranged in a perfectly radial and a random geometry. Curves showing HVRΔ*K* ^trans^ *versus* distance were produced by averaging the *K* ^trans^ gradients of all voxels in the high value ROIs. **Figure 5G** illustrates that the HVRΔ*K* ^trans^ *versus* distance curves differed for the three geometries.

**Figure 5H** shows HVRΔ*K* ^trans^ *versus* distance curves for all untreated and sunitinib-treated BxPC-3 tumors and all untreated and sunitinib-treated Panc-1 tumors included in the study. The parameter $\text{HVR}\Delta K_{2\text{ mm}}^{\text{trans}}$ was extracted from the curves to quantify intratumor heterogeneity, and $\text{HVR}\Delta K_{2\text{ mm}}^{\text{trans}}$ was higher for untreated BxPC-3 tumors than for untreated Panc-1 tumors (**Figure 5I**; *P* = 0.0004). Untreated BxPC-3 tumors showed higher $\text{HVR}\Delta K_{2\text{ mm}}^{\text{trans}}$ than sunitinib-treated BxPC-3 tumors (**Figure 5I**; *P* = 0.0022), whereas untreated and sunitinib-treated Panc-1 tumors did not differ in $\text{HVR}\Delta K_{2\text{ mm}}^{\text{trans}}$ (**Figure 5I**; *P* > 0.05). **Figure 5J** shows $\text{HVR}\Delta K_{2\text{ mm}}^{\text{trans}}$ *versus* median *K* ^trans^ for individual tumors, illustrating that the $\text{HVR}\Delta K_{2\text{ mm}}^{\text{trans}}$ values were not related to the median *K* ^trans^ values and thus provided additional information to the median *K* ^trans^ values.

**Figure 6:**
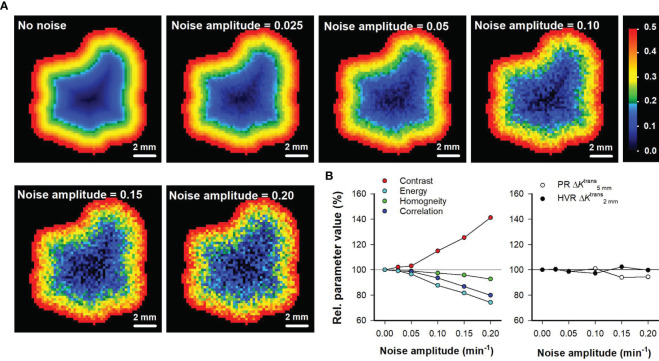
**(A)***K*^trans^ maps of the representative untreated BxPC-3 tumor shown in **Figure 1 ** when the tumor voxels have been rearranged in a perfectly radial geometry without added noise, and with random noise added. The random noise was created by using increasing noise amplitudes as indicated in the maps. The *K* ^trans^ scale is given by the color bar. **(B)** Relative parameter values of the Haralick features Contrast, Energy, Homogeneity, and Correlation *versus* noise amplitude, and relative parameter values of the intratumor heterogeneity parameters $\text{PR}\Delta K_{5\text{ mm}}^{\text{trans}}$ and $\text{HVR}\Delta K_{2\text{ mm}}^{\text{trans}}$ *versus* noise amplitude. The relative parameter values refer to % of the parameter value in the map without added noise. Solid horizontal lines indicate 100% (no change).

The Haralick features and the parameters derived by the spatial gradient method consider the spatial location of the individual voxels. To investigate how these intratumor heterogeneity parameters are influenced by imaging noise, random noise with increasing amplitudes was added to *K* ^trans^ maps after the tumor voxels had been rearranged in a perfectly radial geometry. *K* ^trans^ maps rearranged in a perfectly radial geometry were used because these only showed radial heterogeneity and no random variation, implying that the level of noise in the maps could be controlled by the level of random noise that was added to the maps. **Figure 6A** shows *K* ^trans^ maps of the representative untreated BxPC-3 tumor without added noise and with random noise with increasing amplitudes added to the maps. The Haralick features Contrast, Energy, Homogeneity, and Correlation were found to be strongly influenced by the random noise. Thus the parameter values calculated from maps with added noise differed from the values calculated from the map without noise, and the deviations increased with increasing noise amplitudes (**Figure 6B**). In contrast, the parameters derived by the spatial gradient method ($\text{PR}\Delta K_{5\text{ mm}}^{\text{trans}}$ and $\text{HVR}\Delta K_{2\text{ mm}}^{\text{trans}}$) were similar independent of whether they were calculated from the map without noise or whether they were calculated from maps with added noise (**Figure 6B**). These observations imply that $\text{PR}\Delta K_{5\text{ mm}}^{\text{trans}}$ and $\text{HVR}\Delta K_{2\text{ mm}}^{\text{trans}}$ are less influenced by random noise than the Haralick features.

## Discussion

We chose to evaluate the performance of the spatial gradient method in PDAC because PDACs are particularly aggressive and resistant to therapy (20–22). In addition, PDACs show substantial inter- and intratumor heterogeneity in the physiological tumor microenvironment, suggesting that the aggressive phenotype may be a result of highly heterogenous lesions (21, 26). The microenvironment of PDACs is characterized by a heterogeneous vessel distribution (21), regions with hypoxic tissue (22), and a dense and extensive collagen-rich stroma (22). Low microvascular density has been associated with tumor aggressiveness (27–29), and high microvascular density in peripheral hot spots has been found to correlate with poor prognosis, suggesting that highly heterogeneous vascular supply worsen prognosis (21, 30). It has also been revealed that poor outcome is associated with severe hypoxia in the primary tumor (31, 32). Moreover, it has been suggested that a dense and extensive stroma may cause resistance to treatment by inhibiting the distribution of chemotherapeutic agents (26).

Data sets of two PDAC models were used in the current study, and both untreated and sunitinib-treated tumors were investigated. The data sets were chosen because they showed substantial variation in intratumor heterogeneity. Thus untreated BxPC-3 tumors showed a higher range of *K* ^trans^ values and a more pronounced radial heterogeneity than untreated Panc-1 tumors. Furthermore, sunitinib-treated BxPC-3 tumors did not display radial heterogeneity and showed a lower range of *K* ^trans^ values than untreated BxPC-3 tumors, whereas sunitinib-treated Panc-1 tumors did not differ from untreated Panc-1 tumors in radial heterogeneity or *K* ^trans^ range. The biology behind the differences in *K* ^trans^ between BxPC-3 and Panc-1 tumors has been described previously (23, 33, 34). Importantly, we have shown BxPC-3 and Panc-1 tumors have several microenvironmental features in common with human PDACs, including a histological appearance characterized by a dense collagen-rich extracellular matrix (34, 35). Furthermore, the numerical values of microvascular density and the hypoxic tumor fraction of BxPC-3 and Panc-1 tumors are similar to those reported for human PDACs (35), implying that BxPC-3 and Panc-1 tumors should be highly relevant models for evaluating the performance of the spatial gradient method.

The data sets were also chosen because they showed low levels of imaging noise. DCE-MRI was performed on a state-of-the-art 7-T preclinical scanner, and MR-sequences with a relatively low time resolution were chosen to ensure a high signal-to-noise ratio. In addition, the tumor-bearing mice were anaesthetized and fixed during imaging to minimize motion that could generate imaging noise. Importantly, we demonstrated that the level of noise in the DCE-MRI series was sufficiently low to produce good pharmacokinetic model fits for individual voxels with both high and low uptake of contrast agent, in all tumor groups. It is highly advantageous to use data sets with low levels of imaging noise because noise can conceal small differences in biological variation.

To evaluate novel and established methods to quantify intratumor heterogeneity, a strategy of three steps was used. First, parameters derived by the methods were compared with the ground truth. Thus, we investigated whether the parameters were able to detect tumor line differences as well as treatment-induced changes in *K* ^trans^ range and the radial heterogeneity assessed by concentric ROIs. Second, the parameters were compared with the median *K* ^trans^ to evaluate whether the parameters provided additional information to the median, and third, the sensitivity of the parameters to imaging noise was investigated by adding random noise to *K* ^trans^ maps.

The spatial gradient method was used to measure gradients in *K* ^trans^ maps, and the parameters derived from the gradient curves were sensitive to tumor line differences as well as treatment-induced changes. Importantly, the parameters provided additional information to the median, and were not substantially altered when random noise was added to the *K* ^trans^ maps. Spatial gradients were measured from both peripheral and high value ROIs. Gradients measured from peripheral ROIs are highly useful for describing the heterogeneity in tumors with a radial geometry, whereas gradients measured from high value ROIs may be used to measure intratumor heterogeneity also in tumors with a more complex geometry.

The intratumor heterogeneity parameters $\text{PR}\Delta K_{5\text{ mm}}^{\text{trans}}\text{ and}$ $\text{HVR}\Delta K_{2\text{ mm}}^{\text{trans}}$ were measured at a distance of 5 mm from the peripheral ROIs and at a distance of 2 mm from the high value ROIs respectively. These distances are similar to the radius and half the radius of the tumors included in the study. In tumors with a different size, it may be beneficial to measure at other distances. However, the spatial gradient curves of the tumor groups presented here differed for a large range of distances, implying that it is not crucial to find the optimal distance to detect tumor line differences or treatment-induced changes in intratumor heterogeneity.

None of the parameters derived by histogram analyses considered the spatial location of the individual voxels, and consequently, the histogram-derived parameters were insensitive to differences in radial heterogeneity. The variance and the histogram width mirrored the *K* ^trans^ range in untreated and sunitinib-treated tumors, but the parameters were strongly correlated to the median *K* ^trans^, questioning whether the parameters provided additional information to the median. The histogram skewness differed between untreated BxPC-3 and Panc-1 tumors but was insensitive to sunitinib-induced effects. Thus, the histogram analyses provided less information on intratumor heterogeneity than the spatial gradient method.

The Haralick features Contrast and Energy were higher in Panc-1 tumors than in BxPC-3 tumors. However, these findings were not consistent because the higher Contrast implied that Panc-1 tumors were more heterogeneous than BxPC-3 tumors whereas the higher Energy implied that Panc-1 tumors were more homogeneous than BxPC-3 tumors (18). Furthermore, none of the Haralick features were sensitive to the sunitinib-induced effects, and we demonstrated that the Haralick features were severely influenced by random noise. The data sets used in the current study have low levels of imaging noise, but despite this, the Haralick features were unable to detect tumor-line differences and treatment-induced changes in intratumor heterogeneity. It is thus unlikely that the Haralick features can be used to quantify intratumor heterogeneity in DCE-MRI-derived images of human tumors obtained in unanaesthetized patients using standard clinical scanners where the level of imaging noise is expected to be substantially higher than in the data sets used here. Interestingly, the spatial gradient method was less influenced by random noise and superior to the Haralick features for quantifying intratumor heterogeneity in untreated and sunitinib-treated xenografts, suggesting that the spatial gradient method may be applied in DCE-MRI-derived images of human tumors also.

In the current study, the spatial gradient method was used to measure intratumor heterogeneity in *K* ^trans^ maps. *K* ^trans^ is generally determined by the blood perfusion and the vessel permeability surface-area product (19, 36), but in tumors with high vessel permeability, the uptake of small-molecular-weight contrast agents has been shown to be limited by the blood perfusion rather than the vessel permeability (37–39). We have previously found strong correlations between median *K* ^trans^ and vessel density and between median *K* ^trans^ and hypoxic fraction in BxPC-3 and Panc-1 tumors, implying that *K* ^trans^ reflected blood perfusion in these models (23, 34). The spatial gradient method may also be used to quantify intratumor heterogeneity in other medical images sensitive to blood perfusion and hypoxia, including maps derived by diffusion-weighted MRI (DW-MRI), blood oxygen level dependent MRI (BOLD), tissue oxygen level dependent MRI (TOLD-MRI), and oxygen-enhanced MRI (OE-MRI) (40–42), as well as images derived by dynamic computed tomography (D-CT) and positron emission tomography (PET) (43). The biological interpretation of intratumor heterogeneity as well as the level of imaging noise may differ markedly for the different imaging modalities and should be investigated carefully in novel studies.

The spatial gradient method may also be used to assess changes in intratumor heterogeneity induced by others treatments than sunitinib. For treatments targeting tumor angiogenesis and tumor vasculature such as treatments with antiangiogenic drugs or vascular disrupting agents (44, 45), it may be beneficial to assess intratumor heterogeneity in *K* ^trans^ maps. For treatments targeting tumor cells without affecting the tumor vasculature, it may be more beneficial to assess intratumor heterogeneity in other medical images (1). By assessing treatment-induced changes in intratumor heterogeneity, tumor subregions that respond differently to the treatment may be identified. Detection of poorly responding regions can be important because these regions may repopulate the tumor even if the treatment completely eradicates the tumor mass in other regions (1, 3).

In summary, the spatial gradient method presented in this communication was sensitive to tumor line differences as well as treatment-induced changes in spatial heterogeneity, provided additional information to the median value, and was not confounded by random noise. In contrast, Haralick features were highly sensitive to random noise and insensitive to spatial heterogeneity even in data sets with low level of imaging noise. Histogram analyses did not provide information on spatial heterogeneity, but provided information on the range of *K* ^trans^ values. However, the parameters derived by histogram analyses were strongly correlated to median *K* ^trans^ and thus did not provide additional information to the median. In conclusion, the spatial gradient method was superior to the Haralick method and histogram analyses for quantifying spatial heterogeneity in *K* ^trans^ maps of untreated and sunitinib-treated tumors, and can possibly be used to quantify spatial heterogeneity in other medical images and to evaluate effects of other treatments as well.

## Data Availability Statement

The raw data supporting the conclusions of this article will be made available by the authors, without undue reservation.

## Ethics Statement

The animal study was reviewed and approved by the Institutional Committee on Research Animal Care, Department of Comparative Medicine, Oslo University Hospital, Norway and the Norwegian Food Safety Authority (Mattilsynet), Brumunddal, Norway.

## Author Contributions

J-VG and ER conceived and designed the study, analyzed, and interpreted the data. J-VG wrote the manuscript. All authors contributed to the article and approved the submitted version.

## Funding

Financial support was received from the Norwegian Cancer Society and the South-Eastern Norway Regional Health Authority.

## Conflict of Interest

The authors declare that the research was conducted in the absence of any commercial or financial relationships that could be construed as a potential conflict of interest.

## Publisher’s Note

All claims expressed in this article are solely those of the authors and do not necessarily represent those of their affiliated organizations, or those of the publisher, the editors and the reviewers. Any product that may be evaluated in this article, or claim that may be made by its manufacturer, is not guaranteed or endorsed by the publisher.
